# Perceptions of Weight Change Among Romantic Partners: Considering Body Image, Relationship Experiences, Gender, and Sexual Orientation

**DOI:** 10.3389/fgwh.2022.798257

**Published:** 2022-05-20

**Authors:** Charlotte H. Markey, Kristin J. August, Kristin Kelly, Jamie Price Dunaev

**Affiliations:** Department of Psychology and the Health Sciences Center, Rutgers University–Camden, Camden, NJ, United States

**Keywords:** weight perceptions, body image, romantic relationships, romantic partners, gay partners, lesbian partners

## Abstract

Romantic relationship experiences have been found to be relevant to body image and weight in adulthood. In this study, we investigated predictors of heterosexual, lesbian, and gay romantic partners' (*N* = 500, *M*_*age*_ = 29.3) perceptions of their own and their partners' weight at the beginning of their relationship and 4.8 years later, on average. Perceived changes in *participants'* own weight status was associated with greater body dissastisfaction and longer relationship length. Perceived changes in *partners'* weight status was associated with their partners' BMI, as well as relationship quality. We also found that gender was important in understanding some of these associations. Implications of weight perceptions for individuals' and their partners' health and well-being and the critical role of relationship quality are discussed in the context of the health regulation model.

## Introduction

Individuals' perceptions of their body and weight are believed to be socioculturally constructed. In other words, how people perceive their bodies has only limited association with more objective assessments of their bodies including their actual anthropometric measurements and even others' perceptions of their bodies [e.g., (1)]. This paper focuses on body weight perceptions because some research indicates that perceptions are more predictive of health attitudes and behaviors than one's objective body weight [e.g., eating habits are affected by perceived weight; (2)]. Perceptions of one's partner's weight are also important as partners may be ideal sources of support in the introduction and maintenance of relevant, positive health habits (3). There is no research to date examining partners' perceptions of each other's weight statuses, however.

In addition to the positive benefits being in a romantic relationship may confer to one's health (3), being involved in a romantic relationship might also lead to changes to health habits that result in weight gain. Indeed, evidence suggests that body size is influenced not only by individuals' genes, but also by a number of social factors, one of which is marital status (4). The present study examined individuals' perceptions of their own and their (heterosexual, gay, and lesbian) partners' weight status retrospectively at the start of their relationship and at the time of data collection (on average, 4.8 years after the start of their relationship). In this study, we examine the associations between individuals' and their partners' perceptions of their own and each other's weight change in association with actual weight status and potential associations with body image, relationship factors, age, gender, and sexual orientation.

## The Role of Romantic Partners in Understanding Weight Change in Adulthood

Most people tend to gain weight as they age; research shows most people gain nearly 10 pounds per decade starting in their 20s. For most adults, this trend continues through midlife until they reach their 60s, at which point they may begin to lose weight (5). Although popular cultural perceptions of weight-related concerns indicate that teenagers primarily experience body dissatisfaction, research suggests that body dissatisfaction often persists into adulthood and even beyond middle age; approximately 50% of women and up to 25% of men experience body dissatisfaction (6, 7). Adulthood is also a time when most people tend to develop long-term romantic partnerships, with approximately 55% of American adults between the ages of 18 and 34 years old reporting that they are in a committed romantic relationship (8). The trend for married individuals to weigh more than their unmarried peers was first empirically discussed in research by Sobal et al. (4, 9). In this research, Sobal (4, 9) explored how marriage could change social roles and time commitments. For example, people may move in with their partner and change their eating or physical activity habits (4). Sobal (4) also explored how these changes differed by gender, with women gaining more weight when married, as compared to men, due to gender norms [e.g., appearance and body image concerns may affect women more when they are single; (9)].

There are many theories as to why marriage (or a committed partnership) may contribute to weight gain. For example, the “mating market model” suggests that people in relationships feel safe, secure, and are not “on the market,” so they are less concerned with maintaining sociocultural ideals of attractiveness, which includes thin body ideals for women and lean, muscular ideals for men (4, 10). This model has sustained support in recent research, including a study where both men and women experienced significant weight gain after 4 years or longer in their relationship (11). Other studies have similarly shown how transitions into committed relationships, such as marriage, are related to increases in BMI and decreases in health promoting behaviors [e.g., (12, 13)].

An alternative theory to explain weight changes within romantic relationships is the “health regulation model” (14). This model posits that individuals in more satisfying relationships experience more support and less stress, which benefits their health. Previous research examining the health regulation model suggests that higher relationship quality may protect partners from weight change (14, 15). Recent research, however, indicates that relationship quality is not necessarily associated with positive health behaviors—including eating and physical activity habits—as this model suggests (10, 11, 16). One such study revealed that couples with greater relationship satisfaction were more likely to gain weight over the course of the relationship, supporting the mating market model (10). Another study noted a gender difference, with marital quality being positively associated with women's perceptions that their weight was an issue of concern in their relationship and negatively associated with men's perception that their weight was an issue of concern in their relationship (17). Additional research is needed to clarify how *relationship quality* and weight are associated among romantic partners.

Relationship quality is only one facet of relationships that may affect individuals' health. *Relationship length* indicates, at least, in part, the level of commitment in a relationship. Although research examining the association between relationship length and weight perceptions does not exist, there are data on the association between body image and relationship length. For example, in a study investigating body image among heterosexual couples, relationship length was related to body image for young women, with women in longer relationships experiencing more body dissatisfaction (1). The results from this study indicated that women were more dissatisfied with their own bodies than men and they also overestimated their partner's dissatisfaction with their bodies to be greater than it actually was (this study doesn't address men's body dissatisfaction). In fact, it has been posited that body image is a “couple” variable that is shaped, in part, by one's relationships—especially romantic partners—and is influenced by social comparisons made to one's partner (18, 19).

## LGBTQ+ Partners, Body Image, and Weight

Most research examining relationships and health has focused on heterosexual couples. However, there is emerging research exploring relationships and health—and specifically weight and body image—among individuals in LGBTQ relationships. For example, among gay men, studies suggest a heightened concern about weight and body image and elevated risk for disordered eating (20–23). In the context of relationships, gay men tend to regulate their partners' eating and health behaviors more than heterosexual men, heterosexual women, or lesbian women (24). Gay men also report greater concern about losing physical attractiveness and bodily function as they age (21, 25) and nearly one-third of gay men have experienced negative judgments from other gay men about their body size (26).

In contrast to gay men, lesbian women may be protected from the standard beauty ideals that encourage thinness among women, perhaps due to less sexual objectification in lesbian subculture (27). In a meta-analysis by Morrison et al. (22), lesbian women reported greater body satisfaction than heterosexual women or gay men. Both heterosexual and lesbian women have been found to be less concerned with their partners' thinness or attractiveness as compared to gay men and heterosexual men (28). Some research, however, has found that lesbian women report concerns about thinness that compare to heterosexual women's concerns (29, 30). This study will add to this research by further considering the role of body image, individuals' gender and their partners' gender (i.e., sexual orientation) in perceptions of weight gain in relationships.

## The Current Study

Given research indicating that individuals tend to gain weight across adulthood, especially in the context of relationships, and that weight gain may present some physical and mental health risks (i.e., body dissatisfaction) and be associated with health behaviors (i.e., eating and activity habits), this study aimed to examine possible predictors of individuals' and their partners' perceptions of changes in weight across their relationship. The first aim of this study was to determine if the difference between individuals' perceptions of their current weight and weight at the start of their relationship was associated with their actual weight (BMI), body satisfaction, relationship quality, and relationship length. Individuals' gender and age were considered as covariates and gender was also examined as a potential moderator of the association between each predictor variable and perceived weight change. Additionally, the interaction between participants' gender and their partners' gender (i.e., sexual orientation) was considered as a potential moderator of the association between each predictor variable and perceived weight change.

The second aim of this study was to determine if the difference between individuals' perceptions of their *partners'* current weight and weight at the start of their relationship were associated with their actual weight (participants' BMIs), their partners' BMI, satisfaction with their partners' body, relationship quality, and relationship length. Individuals' gender and age were considered as covariates and gender also was examined as a potential moderator of the association between each predictor variable and perceived partner weight change. Additionally, sexual orientation was considered as a potential moderator of the association between each predictor variable and perceived partner weight change.

## Methods

### Participants

A total of 500 men and women (250 couples) participated in this study of romantic relationships and health. Two hundred and twelve adults in heterosexual relationships (106 women, *M*_*age*_ = 23.87 years; 106 men, *M*_*age*_ age = 25.88 years) and two hundred and eight adults in same-gender relationships (72 couples self-identified as gay: *M*_age_ = 34.1 years; 72 couples self-identified as lesbian: *M*_age_ = 33.3 years years). Couples were required to have been together romantically for a minimum of 6 months and to come to the lab with their partner to participate in the study. The average relationship length for all couples was 4.8 years (*SD* = 6.6 years). Exclusion criteria limited participation to individuals currently without serious, chronic health problems or any health issues (e.g., diabetes) that affected their eating behaviors. (If participants engaged in disordered eating behaviors but did not conceptualize them as disordered, they may have participated).

The participants in heterosexual relationships were predominantly European American (72% European American, 10% African American, 8% Hispanic/Latino, 7% Asian American, and 3% “other”). Participants reported individual incomes in ranges: “ < $20,000” (63%), “$20,000–49,000” (26%), “$50,000–75,000” (10%), and “>$75,000” (1%). Additionally, 41.1% reported that they were dating and not cohabitating, 32.4% reported that they were cohabitating (living with each other), and 26.5% reported that they were married.

The participants in gay and lesbian relationships were also predominantly European-American (70%; 14% African American, 10% Hispanic/Latino, 3% Asian American, 3% “other”). Participants reported individual incomes in ranges: “ < $20,000” (27%), “$20,000–49,000” (36%), “$50,000–75,000” (18%), “$76,000–99,000” (9%), and “100,000 or greater” (10%). Additionally, 83.1% reported that they were cohabitating When these data were collected, same-gender marriage was not legal. The majority of same-gender couples indicated that they would like to be legally married (67%), some indicated “perhaps, someday” (28%), and only 5% indicated “no.” None of the heterosexual couples had children and only a minority of the gay (*N* = 2) and lesbian (*N* = 18) couples were parents.

### Procedure

Participants were recruited with advertisements in the Philadelphia, PA and Camden, NJ (USA) metro-areas and couples were compensated for their time. Once recruited, each romantic partner independently completed an in-person survey in the PI's lab. Consent forms were completed by all participants and all methods were approved by the Institutional Review Board where the research took place.

### Measures

#### Perceived Weight Status

Perceptions of weight change were measured using the Partner Feeding Questionnaire [see (31)], which was adapted from the Child Feeding Questionnaire, a measure that has been used in thousands of studies examining parents' perceptions of their children's weight (32). As the study design was cross-sectional, participants were asked to retrospectively estimate their weight at the start of their relationship, as well as estimate their current weight at the time of data collection. Participants were asked what they believed their own weight and their partners' weight was at the beginning of their relationship, on a 5-point Likert scale from: (1) “markedly underweight”; (2) “underweight”; (3) “normal”; (4) “overweight”; and (5) “markedly overweight” (31). Then, participants were asked what they believed their own weight and their partners' weight was currently (at the time of data collection) on the same 5-point Likert scale. Perceptions from these two time points were used to create a discrepancy score of perceived weight change (current perceived weight—perceived weight at the beginning of the relationship). If perceptions of current and early relationship weights were the same, this would result in a score of 0, with perceptions of weight gain resulting in positive values and perceptions of weight loss resulting in negative values. Given research indicating the importance of weight perceptions [e.g., (2)]—not just objective, measured weight status—this was intended to be a broad assessment of weight perceptions; a subjective assessment of participants and their partners. Because these items are not necessarily expected to be interrelated, we do not report internal consistency reliability; past research has found this measure to have predictive validity (31, 32).

#### Body Mass Index

Body mass index (BMI) was computed using researchers' measurements of participants' height in centimeters using a stadiometer and weight in kilograms via a standard medical scale. Weight and height were recorded three times for each participant and the average of the three measures was used [e.g., (19)]. Romantic partners tended to have fairly similar BMIs [intraclass *r* (250) = 9.38, *p* < 0.001].

#### Body Image

To assess satisfaction with individuals' own, and their partners,' bodies, the Contour Drawing Rating Scale [CDRS; (33)] was used. Participants were asked to select 1 of 9 gender-specific figures (1 = *very underweight*, 9 = *very overweight*) that represented: (1) what they think they currently look like (i.e., current body), (2) what they would like to look like (i.e., ideal body), (3) what they think their romantic partner currently looks like (i.e., views of their partner's current body), and (4) what they would like their partner to look like (i.e., views of their partner's ideal body). From these items, two body satisfaction scores were calculated: Participants' *own body dissatisfaction* (calculated by subtracting item #1 from item #2) and participants' *dissatisfaction with their partners' bodies* (calculated by subtracting item #3 from item #4). Absolute values of these scores were crated so that a score of 0 indicates satisfaction and a score > 0 indicates some level of dissatisfaction. The test–retest reliability for this measure has been reported to be good [0.79; (33)], and this measure has been found to have predictive validity [e.g., it is associated with weight status and dieting behaviors in other adult samples; (34, 35)].

#### Relationship Quality

The Marital Interactions Questionnaire [MIS; (36)] was used to assess participants' relationship quality. This 15-item measure contains two subscales of love and conflict. The love scale queried participants using ten items including “How committed do you feel toward your partner?” The conflict scale queried participants using five items including “How often do you and your partner argue with one another?” Each item was rated on a 9-point Likert scale from 0 = “not at all” to 8 = “very much.” Items assessing conflict were reverse coded (thus assessing “harmony”) and a total composite score of relationship quality was computed. The original format of the MIS was designed to assess married couples' relationship quality; the measure was revised for this study to read “significant other” and “partner,” rather than “spouse.” The MIS was reliable across the subsamples of couples (αs = 0.77–0.89) and has been used successfully in other studies of same-sex couples, revealing predictive validity [e.g., (19)].

#### Relationship Length

Participants reported their relationship length by answering: “For how many months have you been continuously involved with your romantic partner?” Answers are presented in years for easier interpretation.

#### Covariates

Analyses considered covariates likely associated with weight gain and relationship status: age, participants' gender (coded −1 = male, 1 = female), and sexual orientation. (Gender and sexual orientation also were considered as moderators in analyses).

### Analytic Plan

SPSS version 28 and HLM version 7 were used for descriptive analyses and to test study aims. Data were checked for completeness; the amount of missing data on any variable used in analyses ranged from 0 to 1.4%. Listwise deletion was used for missing data. To account for the non-independence of data from individuals nested within relationships, multilevel modeling was used to test for gender and sexual orientation differences in within-couples variables and to test study aims (37). Continuous variables were group mean centered at level 1 (within-couples) and grand mean centered at level 2 (between-couples).

First, descriptive analyses were conducted to examine gender and sexual orientation differences in key study variables. For the between-couples variable (relationship length), a one-way ANOVA was conducted to examine sexual orientation differences. For within-couples variables, individuals' gender was examined as a predictor in multilevel models to determine gender differences; individuals' gender, their partners' gender, and the interaction between the two, were examined as predictors in multilevel models to determine sexual orientation differences.

Next, study aims were examined using the factorial method (38), an extension of the Actor Partner Interdependence Model, as couples were both indistinguishable (same-gender relationships) and distinguishable (different-gender relationships). This method produces multilevel regression estimates for four groups: heterosexual men, heterosexual women, gay men, and lesbian women. The multilevel models were analyzed using full maximum likelihood; in addition, because dyadic analyses limit the number of random-effects parameters that can be estimated, random slopes were not estimated.

Six multilevel regression models were conducted to examine the study aims. In the first set of three models, BMI, body dissatisfaction, relationship quality, relationship length, gender, and age were examined as predictors of participants' perceptions of their own weight change (Model 1). Second, interactions between participants' gender with BMI, body dissatisfaction, relationship quality, relationship length, and age were examined as predictors of participants' perceptions of their own weight change (Model 2). Third, the interaction between participants' gender and their partners' gender (i.e., sexual orientation) was added to the model to determine the extent to which sexual orientation moderated associations between each predictor and participants' perceptions of their own weight change (Model 3). (Note: interactions with partner gender also were included in this model, but those results are not presented because they do not address our study aims but are still needed before testing actor gender^*^partner gender.)

The second set of three models examined participants' perceptions of their *partners*' weight change, first considering the main effects of participants' BMI, their partners' BMIs, dissatisfaction with their partners' bodies, relationship quality, relationship length and the covariates age and gender (Model 1). Next, interactions between participants' gender with their BMI, partners' BMI, dissatisfaction with their partners' bodies, relationship quality, relationship length, and age were considered in predicting participants' perceptions of their *partners'* weight change (Model 2). Finally, the interaction between participants' gender and their partners' gender (i.e., sexual orientation) was added to the model to determine the extent to which sexual orientation moderated associations between each predictor and participants' perceptions of their partners' weight change (Model 3). (Again, interactions with partner gender were also included in this model because they are needed before testing actor gender^*^partner gender.) Prior to creating interactions with gender, the variables were grand mean-centered. The *t* statistics from the multilevel models were transformed into partial correlations to provide a measure of effect size [$pr=\sqrt{t^{2}/\left(t^{2}+df\right)}$; (39)].

## Results

Table 1 presents the means and standard deviations for the key study variables by gender and sexual orientation. Only two significant actor gender differences emerged: Women were more likely than men to be dissatisfied with their own bodies and also were more likely to be dissatisfied with their partners' bodies. There were significant partner gender differences in BMI, such that individuals who had a partner who was a male (i.e., heterosexual women and gay men) had lower BMIs than those who had a partner who was a female (i.e., heterosexual men and lesbian women). There were also sexual orientation differences in relationship length, BMI, and relationship quality. *Post-hoc* tests were not significant, however, for relationship length. For BMI, gay and lesbian couples overall had higher BMIs than heterosexual couples, with the largest difference seen between lesbian women and heterosexual women. Finally, for relationship quality, gay men and lesbian women had significantly higher relationship quality than heterosexual men and women, with the largest difference seen between lesbian women and heterosexual women.

**Table 1:** Descriptive statistics for key study variables by gender and sexual orientation.

	**Heterosexual men** **(*****n*** **=** **106)**			**Heterosexual women** **(*****n*** **=** **106)**			**Gay men** **(*****n*** **=** **144)**			**Lesbian women** **(*****n*** **=** **144)**			**Participant gender** **differences**	**Partner gender** **differences**	**Sexual orientation** **differences**
	**Range**	***M***	***SD***	**Range**	***M***	***SD***	**Range**	***M***	***SD***	**Range**	***M***	***SD***			
**Between-couples variable**															
Relationship length (years)	1.0–35.0	3.89	4.59	1.0–35.0	3.89	4.59	0.50–61.50	6.41	9.94	0.50–19.00	4.69	4.47	_________	________	**3.20 (2)** ^*^ ^a^
**Within-couples variables**															
BMI	18.79–49.66	27.46	5.96	17.45–48.59	24.27	5.56	16.89–40.92	26.28	4.59	16.88–61.83	29.38	8.23	−0.41 (0.28)	1.57 (0.28)^***^	0.98 (0.33)^**^
Dissatisfaction with own body	0–3.0	1.02	0.92	0–6.0	1.58	1.37	0–4.0	1.31	1.05	0–6.0	1.71	1.09	0.24 (0.05)^***^	−0.04 (0.05)	0.10 (0.05)
Dissatisfaction with partner's body	0–4.0	0.93	0.92	0–8.0	1.24	1.45	0–4.0	0.99	1.03	0–5.0	1.25	1.13	0.12 (0.05)^*^	−0.03 (0.05)	0.03 (0.05)
Relationship quality	8.50–16.60	13.04	1.84	5.20–16.60	12.90	2.03	8.80–17.60	13.69	1.57	6.80–17.70	13.86	1.82	−0.02 (0.08)	0.08 (0.08)	0.40 (0.10)^***^
Weight change: self	−1.0 to 3.0	0.30	0.62	−1.0 to 4.0	0.43	0.77	−2.0 to 3.0	0.31	0.79	−2.0 to 3.0	0.36	0.83	0.04 (0.03)	−0.02 (0.03)	−0.01 (0.04)
Weight change: partner	−1.0 to 3.0	0.22	0.59	−2.0 to 2.0	0.19	0.57	−2.0 to 3.0	0.30	0.72	−2.0 to 2.0	0.17	0.69	−0.04 (0.03)	−0.03 (0.03)	0.02 (0.03)

For the between-couples variable, sexual orientation differences were examined using sexual orientation as a predictor in a one-way ANOVA; the value presented is an F statistic (degrees of freedom). For the within-couples variable, participant gender, partner gender, and sexual orientation (participant gender ^*^ partner gender) differences were examined using actor gender, partner gender, and actor ^*^ partner gender as predictors in multilevel models; values presented are unstandardized estimates (standard error). Significant gender or sexual orientation differences are shown in bold.

a *Post-hoc tests did not reveal significant differences*.

* *p < 0.05;*

** *p < 0.01;*

*** *p < 0.001*.

### Associations With One's Own Perceived Weight Change

Our first aim was to examine predictors of participants' perceptions of their own weight change. To test for main effects, participants' BMI, body dissatisfaction, relationship quality, relationship length, age, and gender were tested as predictors of participants' own perceived weight change. Results (Table 2) revealed that participants' body dissatisfaction and relationship length significantly predicted participants' perceptions of their own weight change. Specifically, participants with greater dissatisfaction with their bodies and in longer relationships perceived a significant increase in their own weight from the start of their relationship until the time they participated in this study. In examining participants' gender and sexual orientation (participants' gender ^*^ partners' gender) as potential moderators, one significant interaction with actor gender emerged. Simple slopes analysis revealed that for men, having a higher BMI was associated with a greater amount of perceived weight change [simple slope = 0.02 (0.01), *t* = 2.61, *p* = 0.01], whereas there was no association between BMI and weight change for women [simple slope = −0.01 (0.01), *t* = −0.67, *p* = .50]. There were no significant interactions between sexual orientation (actor gender ^*^ partner gender) with any of the independent variables in predicting individuals' own perceived weight change.

**Table 2:** Multilevel models predicting participants' perceptions of their own weight change (*N* = 500).

	**Estimate**	***SE***	***t***	***p***	**Effect size (*pr*)**
***Model 1: Main effects***					
BMI	<0.01	0.01	0.35	0.73	0.02
**Dissastisfaction with own body**	**0.21**	**0.03**	**6.44**	**<0.001**	**0.39**
Relationship quality	−0.02	0.02	−0.98	0.33	−0.06
**Relationship length**	**0.02**	**0.01**	**3.50**	**<0.001**	**0.22**
Age	< −0.01	<0.01	−1.17	0.24	−0.08
Gender	< −0.01	0.03	<0.01	0.99	< −0.01
***Model 2: Interactions with actor gender***					
BMI*actor gender	**−0.01**	**0.01**	**−2.07**	**0.04**	**−0.13**
Dissastisfaction with own body*actor gender	0.01	0.03	0.38	0.72	0.02
Relationship quality*actor gender	0.02	0.02	1.0	0.32	0.07
Relationship length*actor gender	< −0.01	0.01	−0.31	0.76	−0.02
Age*actor gender	<0.01	<0.01	0.97	0.33	0.06
***Model 3: Interactions with sexual orientation***					
BMI*actor gender*partner gender	<0.01	0.01	0.78	0.44	0.05
Dissatisfaction with own body*actor gender*partner gender	0.03	0.03	1.00	0.32	0.07
Relationship quality*actor gender*partner gender	−0.01	0.02	−0.76	0.45	−0.05
Relationship length*actor gender*partner gender	−0.01	0.01	−0.70	0.48	−0.05
Age*actor gender*partner gender	< −0.01	0.01	−0.79	0.43	−0.05

*Analyses examining interactions with partner gender were included in model 3, but were not part of study aims so are not shown here. Significant findings are shown in bold*.

### Associations With Partners' Perceived Weight Change

Our second aim was to examine predictors of participants' perceptions of their *partners*' weight change. Participants' BMIs, partners' BMIs, dissatisfaction with partners' bodies, relationship quality, relationship length, age, and gender were examined as predictors of perceived partners' weight change. Results (Table 3) revealed that participants' partners' BMI significantly predicted participants' perceptions of their partners' weight change. Specifically, participants whose partners had higher BMIs perceived a significant increase in their partners' weight across the length of the relationship. Relationship quality also had a significant association with perception of partners' weight change, such that participants in higher quality relationships were less likely to perceive an increase in their partners' weight across the length of the relationship. In examining gender and sexual orientation as potential moderators, two significant interactions with actor gender emerged: one with dissatisfaction with one's partners' body and one with relationship length. Simple slopes analysis revealed that for men, greater dissatisfaction with partners' bodies was associated with greater perceived weight change among partners [simple slope = 0.10 (0.03), *t* = 2.92, *p* = 0.004], whereas for women, there was no association between dissatisfaction with partners' bodies and perceived weight change among partners [simple slope = −0.03 (0.04), *t* = −0.81, *p* = 0.42]. In addition, for men, relationship length was not associated with perceived weight change among partners [simple slope ≤ 0.01 (0.01), *t* = 0.12, *p* = 0.91], whereas for women, being in a longer relationship was associated with greater perceived weight change among partners [simple slope = 0.03 (0.02), *t* = 2.49, *p* = 0.01]. There were no significant interactions, however, between sexual orientation (participants' gender ^*^ partners' gender) and any of the independent variables in predicting perceived weight change among partners.

**Table 3:** Multilevel models predicting participants' perceptions of their partner's weight change (*N* = 500).

	**Estimate**	***SE***	***t***	***p***	**Effect size (*pr*)**
*Model 1: Main effects*					
BMI	0.01	<0.01	1.74	0.08	0.11
**BMI partner**	**0.01**	**<0.01**	**2.36**	**0.02**	**0.15**
Dissastisfaction with partner's body	0.02	0.03	0.76	0.45	0.05
**Relationship quality**	**−0.03**	**0.02**	**−1.97**	**<0.05**	**−0.13**
Relationship length	0.01	0.01	1.50	0.14	0.10
Age	<0.01	<0.01	0.52	0.60	0.03
Gender	**−0.06**	**0.03**	**−1.87**	**0.06**	**−0.12**
*Model 2: Interactions with actor gender*					
BMI*actor gender	0.01	0.01	1.10	0.27	0.07
BMI partner*actor gender	< −0.01	0.01	−0.37	0.71	−0.02
Dissastisfaction with partner's body*actor gender	**−0.07**	**0.03**	**−20.44**	**0.02**	**−0.16**
Relationship quality*actor gender	<0.01	0.02	0.28	0.78	0.02
Relationship length*actor gender	**0.01**	**0.01**	**2.08**	**0.04**	**0.14**
Age*actor gender	−0.01	<0.01	−1.68	0.09	−0.11
*Model 3: Interactions with sexual orientation*					
BMI*actor gender*partner gender	<0.01	0.01	0.33	0.74	0.02
BMI partner* actor gender*partner gender	0.01	0.01	1.30	0.19	0.09
Dissastisfaction with partner's body*actor gender*partner gender	−0.01	0.03	−0.50	0.62	−0.03
Relationship quality*actor gender*partner gender	−0.01	0.02	−0.71	0.48	−0.05
Relationship length*actor gender*partner gender	−0.01	0.01	−0.60	0.55	−0.04
Age*actor gender*partner gender	<0.01	0.01	0.53	0.60	0.04

*Analyses examining interactions with partner gender were included in model 3, but were not part of study aims so are not shown here. Significant findings are shown in bold*.

## Discussion

This study examined correlates of individuals' and their partners' perceived changes in their weight from the start of their relationships to the time of data collection (on average, almost 5 years later). We examined BMI (own and partners'), body dissatisfaction (own and partners'), relationship quality, relationship length, age, and gender as predictors of perceived individual and partner weight change and considered gender and sexual orientation as moderators. Findings provided some support for both the “mating market” and “health regulation” models.

Our first aim focused on possible predictors of participants' perceptions of their *own* weight change across their romantic relationship. Results indicated that participants' own dissatisfaction with their bodies and the length of their relationships were significant predictors of perceived weight change. Participants' age was not a significant predictor of their perceived weight change, suggesting that older couples in longer relationships (i.e., who may weight more) do not account for this finding. Of course, it is likely that body dissatisfaction both predicts and is a consequence of weight gain, but future research will need to discern the direction of effects or whether these associations are reciprocal.

Our results also indicated that individuals' perceptions of their own weight change is significantly related to relationship length, but not quality. This finding is consistent with the “mating market model,” where longer relationships, which may represent greater commitment and security, are associated with weight gain. Although relationship support and security confer some health benefits, they may also contribute to a lack of concern about maintaining eating and physical activity patterns that are conducive to health in the long-term (3, 40).

Our examination of moderators of predictors of participants' perceptions of changes in their own weight status revealed one significant interaction: BMI interacted with gender in predicting participants' perceptions of changes in their weight. Among men, having a higher BMI was associated with a greater amount of perceived weight change, but there was no association between BMI and perceived weight change for women. This may be because men's perceptions of their bodies and weight are more grounded in a realistic understanding of their actual body size whereas women's self-perceptions are more heavily influenced by sociocultural ideals of beauty that present unrealistically thin models of attractiveness (6, 41). Additional research that explores weight *perceptions* and the subjective nature of body size perceptions will further our understanding of men's vs. women's experiences of changes in weight during adulthood.

Our second aim was to examine potential predictors of participants' perceptions of changes in their *partners'* weight statuses. We found that partners' actual weight statuses (BMIs) were associated with their perceived changes in weight status. This link between partners' actual weight and perceived change in weight may be expected and suggests that there perceptions were realistic. However, a negative association between participants' perceptions of their partners' weight change and relationship quality was also identified suggesting that social and psychological factors also contribute to these perceptions. Further, this result contributes to research supporting the “health regulation model” (42); higher quality relationships seem to be associated with fewer changes in weight. It is also possible that people may simply find a relationship with a partner who has experienced less weight change more satisfying. Unpacking these directions of effects would be a valuable next step for future research. It would be useful to understand the extent to which concordance in partners' health behaviors may contribute to this finding. For example, do partners who engage in similar levels of physical activity experience less weight gain and also more satisfying relationships?

We also found that gender moderated the association between dissatisfaction with partners' bodies and perceptions of their weight change. Among men, greater dissatisfaction with partners' bodies was associated with greater perceived (partner) weight change. This is consistent with past reports indicating that men (regardless of sexual orientation) value thinness and attractiveness in their partners, whereas this is less of a concern among women (28). It follows then, that among women in this sample, there was no association between dissatisfaction with partners' bodies and perceived weight change among partners.

Gender also moderated the association between relationship length and perceptions of partners' weight change. Among men, relationship length was not associated with perceived weight change among partners, whereas among women, being in a longer relationship was associated with greater perceived weight change among partners. We can only speculate as to the cause of this finding, but it may suggest that women become more attuned to changes in their partner over time but men are less likely to. Past research also suggests that women may be more likely to regulate men's health behaviors than the reverse [e.g., women may assume some responsibility for men's health; (3)], leading women to be more aware of their partners' weight and health habits that may affect weight.

### Limitations

Although we believe this study to be the first to consider predictors of perceptions of weight change within the context of heterosexual, gay, and lesbian partnerships, it is not without limitations. The cross-sectional and correlational design precludes causal interpretations of the data. For instance, although we found that individuals' body dissatisfaction is associated with their perceived change in weight, this might be the product of a reciprocal relationship between perceptions of weight and body dissatisfaction, rather than a sequential outcome. Furthermore, although this sample is somewhat diverse in terms of age and sexual orientation, the sample was limited in diversity in terms of race, ethnicity, and socioeconomic status. We recruited participants who did not have significant, chronic health concerns and who did not have children (although, a small minority of couples did have children), making it impossible to examine the relevance of these factors to our models. Additionally, all participants self-identified as being in a committed heterosexual, lesbian, or gay relationship, but it is possible that participants were bisexual or had maintained different relationships in the past. Thus, future exploration of perceptions of weight change in the context of relationships among a more representative sample is warranted. Finally, we do not have BMI nor body image reports for participants at the initiation of their relationships making it impossible to know how accurate perceptions of weight changes actually are among partners. Given research [e.g., (43)] suggesting the accuracy of self-reported height, weight, and weight status, it seems likely that participants' reports were fairly accurate. Still, future research that had several assessments of romantic partners' weights and perceptions of their own and their partners' bodies prospectively (i.e., from the start of the relationship), using more nuanced assessments of how body and weight change, would be able to better address issues of cause and effect when it comes to partners' body perceptions and weights.

## Conclusion and Implications

Prior research reveals the potential for romantic partnerships to contribute to individuals' health and wellbeing, but also potentially to habits that are not conducive to long-term health (e.g., poor eating habits). The current study adds to the existing literature by highlighting the importance of different relationship factors (length and quality) in understanding romantic partners' perceptions of their own and their partners' changes in weight across their relationship.

These findings may have both theoretical and applied implications. Theoretically, it is important to consider how romantic relationships may affect partners' wellbeing. Although most research suggests that romantic relationships enhance psychological and physical health behaviors and outcomes (3), by contributing to weight gain across time in a relationship, partners may incur health risks such as an increased risk for diabetes and heart disease and possibly mental health concerns [e.g., body dissatisfaction; (44)]. Thus, the health regulation model (42) cannot explain all of the potential associations among relationships and health.

Our findings may also contribute to applied efforts to utilize romantic partners as sources of support when managing health risks as well as chronic and acute health problems [e.g., (45)]. There is very limited research examining LGBTQ couples and partners' role in health behaviors [for exceptions, see research by Garcia and Umberson, e.g., (46)], but our findings suggest that same-sex couples may experience similar relationship dynamics to heterosexual couples when it comes to engagement that has implications for health behaviors and outcomes. It is critical to understand that romantic partners may not always be inclined to support healthy habits and that interventions that include partners may require explicit education and support concerning adaptive body image perceptions, eating, and exercise behaviors (47). By leveraging romantic partners to assist in health behavior and attitudinal changes, it is possible that both individuals' health and relationships will benefit.

## Data Availability Statement

The raw data supporting the conclusions of this article will be made available by the authors upon request.

## Ethics Statement

This study was reviewed and approved by the Rutgers University IRB. The patients/participants provided their written informed consent to participate in this study.

## Author Contributions

All authors listed have made a substantial, direct, and intellectual contribution to the work and approved it for publication.

## Funding

This research was funded by awards from Rutgers University to CM and KA as well as awards from the Lesbian Health Fund of the Gay and Lesbian Medical Association to CM.

## Conflict of Interest

The authors declare that the research was conducted in the absence of any commercial or financial relationships that could be construed as a potential conflict of interest.

## Publisher's Note

All claims expressed in this article are solely those of the authors and do not necessarily represent those of their affiliated organizations, or those of the publisher, the editors and the reviewers. Any product that may be evaluated in this article, or claim that may be made by its manufacturer, is not guaranteed or endorsed by the publisher.

## References

[1] Markey CN Markey PM. Romantic relationships and body satisfaction among young women. J Youth Adolesc. (2006) 35:256–64. 10.1007/s10964-005-9013-6

[2] Haynes A Kersbergen I Sutin A Daly M Robinson E. A systematic review of the relationship between weight status perceptions and weight loss attempts, strategies, behaviours and outcomes. Obesity Rev. (2018) 19:347–63. 10.1111/obr.12634 29266851 PMC5814847

[3] August KJ Kelly CS Markey CN. Marriage, romantic relationships, and health. In: Friedman H, editor. Encyclopedia of Mental Health. 2nd ed. San Diego, CA: Elsevier (2016).

[4] Sobal J. Marriage, obesity and dieting. Marriage Fam Rev. (1984) 7:115–39. 10.1300/J002v07n01_10

[5] Hutfless S Gudzune KA Maruthur N Wilson RF Bleich SN Lau BD. Strategies to prevent weight gain in adults: a systematic review. Am J Prev Med. (2013) 45:e41–51. 10.1016/j.amepre.2013.07.013 24237928

[6] Gillen MM Markey CH. Body image and mental health. In: Friedman HS, Markey CH, editors. Encyclopedia of Mental Health, 3rd ed. New York, NY: Elsevier (2022).

[7] Markey CH August KJ Dunaev J. Understanding body image among adults in mid-late life: considering romantic partners and depressive symptoms in the context of diabetes. J Health Psychol. (2020) 25:1707–16. 10.1177/1359105318770725 29696998

[8] The General Social Survey. Graph Illustration of Trends in Gender and Marriage in the United States, 2018. Solar Spectral Data Access from the GSS (2019). Available online at: https://gssdataexplorer.norc.org/trends/Gender%20&%20Marriage?measure=posslq (accessed October 6, 2021).

[9] Sobal J Rauschenbach BS Frongillo EA. Marital status, fatness, and obesity. Soc Sci Med. (1992) 35:915–23. 10.1016/0277-9536(92)90106-Z 1411692

[10] Meltzer AL Novak SA McNulty JK Butler EA Karney BR. Marital satisfaction predicts weight gain in early marriage. Health Psychol. (2013) 32:824–7. 10.1037/a0031593 23477578

[11] Mata J Richter D Schneider T Hertwig R. How cohabitation, marriage, separation, and divorce influence BMI: a prospective panel study. Health Psychol. (2018) 37:948. 10.1037/hea0000654 30234354

[12] Kutob RM Yuan NP Wertheim BC Sbarra DA Loucks EB Nassir R. Relationship between marital transitions, health behaviors, and health indicators of postmenopausal women: results from the Women's Health Initiative. J Womens Health. (2017) 26:313–20. 10.1089/jwh.2016.5925 28072926 PMC5397241

[13] Josefsson K Elovainio M Stenholm S Kawachi I Kauppi M Aalto V. Relationship transitions and change in health behavior: A four-phase, twelve-year longitudinal study. Soc Sci Med. (2018) 209:152–9. 10.1016/j.socscimed.2018.03.006 29566960

[14] Umberson D Williams K Powers D Liu H Needham B. You make me sick: Marital quality and health over the life course. J Health Soc Behav. (2006) 47:1–16. 10.1177/002214650604700101 16583772 PMC3149975

[15] Chen Y Kawachi I Berkman L Trudel-Fitzgerald C Kubzansky L. A prospective study of marital quality and body weight in midlife. Health Psychol. (2018) 37:247–56. 10.1037/hea0000589 29369677

[16] Skoyen JA Rentscher KE Butler EA. Relationship quality and couples' unhealthy behaviors predict body mass index in women. J Soc Pers Relat. (2018) 35:224–45. 10.1177/0265407516680909

[17] Schade LC Sandberg J Busby D. Does this marriage make me look fat? Marital quality as a predictor of perceptions of body weight, activity level, and eating habits. Am J Family Ther. (2014) 42:42–52. 10.1080/01926187.2013.767617

[18] Markey CN Markey PM. Weight disparities between female same-sex romantic partners and weight concerns: examining partner comparison. Psychol Women Q. (2013) 37:469–77. 10.1177/0361684313484128

[19] Markey CN Markey PM. Gender, sexual orientation, and romantic partner influence on body image: An examination of heterosexual and lesbian women and their partners. J Soc Pers Relat. (2014) 31:162–77. 10.1177/0265407513489472

[20] Bell K Rieger E Hirsch JK. Eating disorder symptoms and proneness in gay men, lesbian women, and transgender and non-conforming adults: Comparative levels and a proposed mediational model. Front Psychol. (2019) 9:2692. 10.3389/fpsyg.2018.02692 30671007 PMC6331421

[21] Lodge AC Umberson D. Age and embodied masculinities: Midlife gay and heterosexual men talk about their bodies. J Aging Stud. (2013) 27:225–32. 10.1016/j.jaging.2013.03.004 23849420 PMC3712281

[22] Morrison MA Morrison TG Sager CL. Does body satisfaction differ between gay men and lesbian women and heterosexual men and women?: a meta-analytic review. Body Image. (2004) 1:127–38. 10.1016/j.bodyim.2004.01.002 18089146

[23] Fichter MM Daser C. Symptomatology, psychosexual development and gender identity in 42 anorexic males. Psychol Med. (1987) 17:409–18. 10.1017/s003329170002496x 3602232

[24] Markey CN Markey PM August KJ Nave CS. Gender, BMI, and eating regulation in the context of same-sex and heterosexual couples. J Behav Med. (2016) 39:398–407. 10.1007/s10865-015-9700-z 26660637

[25] Slevin KF Linneman TJ. Old gay men's bodies and masculinities. Men Masc. (2010) 12:483–507. 10.1177/1097184X08325225

[26] Foster-Gimbel O Engeln R. Fat chance! Experiences and expectations of antifat bias in the gay male community. Psychol Sex Orient Gender Diver. (2016) 3:63–70. 10.1037/sgd0000159

[27] Polimeni AM Austin SB Kavanagh AM. Sexual orientation and weight, body image, and weight control practices among young Australian women. J Womens Health. (2009) 18:355–62. 10.1089/jwh.2007.0765 19281319

[28] Legenbauer T Vocks S Schäfer C Schütt-Strömel S Hiller W Wagner C. Preference for attractiveness and thinness in a partner: influence of internalization of the thin ideal and shape/weight dissatisfaction in heterosexual women, heterosexual men, lesbians, and gay men. Body Image. (2019) 6:228–34. 10.1016/j.bodyim.2009.04.002 19443281

[29] Moreno-Domínguez S Raposo T Elipe P. Body image and sexual dissatisfaction: differences among heterosexual, bisexual, and lesbian women. Front Psychol. (2019) 10:903. 10.3389/fpsyg.2019.00903 31143139 PMC6520663

[30] Smith ML Telford E Tree JJ. Body image and sexual orientation: the experiences of lesbian and bisexual women. J Health Psychol. (2019) 24:1178–90. 10.1177/1359105317694486 28810414

[31] Markey CN Gomel JN Markey PM. Romantic relationships and eating regulation: an investigation of partners' attempts to control each others' eating behaviors. J Health Psychol. (2008) 13:422–32. 10.1177/1359105307088145 18420775

[32] Birch LL Fisher JO Grimm-Thomas K Markey CN Sawyer R Johnson SL. Confirmatory factor analysis of the child feeding questionnaire: a measure of parental attitudes, beliefs, and practices about child feeding and obesity proneness. Appetite. (2001) 36:201–10. 10.1006/appe.2001.0398 11358344

[33] Thompson MA Gray JJ. Development and validation of a new body-image assessment scale. J Pers Assess. (1995) 64:258–69. 10.1207/s15327752jpa6402_6 7722852

[34] Markey C Markey P. Romantic partners, weight status, and weight concerns: An examination using the actor-partner interdependence model. J Health Psychol. (2011) 16:217–25. 10.1177/1359105310375636 21135064

[35] Markey CN Markey PM Gray HF. Romantic relationships and health: an examination of individuals' perceptions of their romantic partners' influences on their health. Sex Roles J Res. (2007) 57:435–45. 10.1007/s11199-007-9266-5

[36] Braiker H Kelley HH. Conflict in the development of close relationships. In: Burgess RL, Huston TL, editors, Social Exchange in Developing Relationships. New York: Academic Press (1979). p. 127–54.

[37] Kenny DA Kashy DA Cook WL. Dyadic Data Analysis. New York, NY: Guilford Press (2006).

[38] West TV Popp D Kenny DA. A guide for the estimation of gender and sexual orientation effects in dyadic data: An actor-partner interdependence model approach. Pers Soc Psychol Bull. (2008) 34:321–36. 10.1177/0146167207311199 18272802

[39] Rosnow RL Rosenthal R. Effect sizes for experimenting psychologists. Can J Exp Psychol. (2003) 57:221–37. 10.1037/h0087427 14596479

[40] Sobal J Hanson KL Frongillo EA. Gender, ethnicity, marital status, and body weight in the United States. Obesity. (2009) 17:2223–31. 10.1038/oby.2009.64 19300431

[41] Grabe S Ward LM Hyde JS. The role of the media in body image dissatisfactions among women: a meta-analysis of experimental and correlational studies. Psychol Bull. (2008) 134:460. 10.1037/0033-2909.134.3.460 18444705

[42] Umberson D. Gender, marital status and the social control of health behavior. Soc Sci Med. (1992) 34:907–17. 10.1016/0277-9536(92)90259-S 1604380

[43] Lipsky LM Haynie DL Hill C Nansel TR Li K Liu D. Accuracy of self-reported height, weight, and BMI over time in emerging adults. Am J Prev Med. (2019) 56:860–8. 10.1016/j.amepre.2019.01.004 31003807 PMC6661894

[44] Centers for Disease Control Prevention. The Health Effects of Overweight and Obesity. (2021). Available online at: https://www.cdc.gov/healthyweight/effects/index.html (accessed January 30, 2022).

[45] Gorin AA Powers TA Gettens K Cornelius T Koestner R Mobley AR. A randomized controlled trial of a theory-based weight-loss program for couples. Health Psychol. (2020) 39:137–46. 10.1037/hea0000808 31789558 PMC6957719

[46] Garcia MA Umberson D. Marital strain and psychological distress in same-sex and different-sex couples. J Marriage Fam. (2019) 81:1253–68. 10.1111/jomf.12582 31496540 PMC6731029

[47] Martire LM Helgeson VS. Close relationships and the management of chronic illness: associations and interventions. Am Psychol. (2017) 72:601–12. 10.1037/amp0000066 28880106 PMC5598776

